# Identification of cholix toxin gene in *Vibrio cholerae* non-O1/non-O139 isolated from diarrhea patients in Bushehr, Iran

**DOI:** 10.18502/ijm.v12i4.3929

**Published:** 2020-08

**Authors:** Marziyeh Gholizadeh Tangestani, Jafar Alinezhad, Abdolmohammad Khajeian, Somayyeh Gharibi, Mohammad Ali Haghighi

**Affiliations:** 1 Department of Microbiology and Parasitology, School of Medicine, Bushehr University of Medical Sciences, Bushehr, Iran; 2 The Persian Gulf Tropical Medicine Research Center, The Persian Gulf Biomedical Sciences Research Institute, Bushehr University of Medical Sciences, Bushehr, Iran; 3 Department of Microbiology, School of Sciences, Kherad Institute of Higher Education, Bushehr, Iran

**Keywords:** *Vibrio cholerae*, Cholix toxin, Polymerase chain reaction

## Abstract

**Background and Objectives:**

Cholixin (cholix toxin) is a novel exotoxin in *Vibrio cholerae* identified as an elongation factor II specific ADP-ribosyltransferase which inhibits protein synthesis in the eukaryotic cell. Previous researches have suggested that cholixin probably is an important virulence factor in non-O1/non-O139 *V. cholerae* (NAG) serotypes that could be related to extra-intestinal rather than intestinal infections. This study was aimed to investigate the frequency and genetic diversity of colixin gene *(chxA)* in clinical *V. cholerae* NAG isolates.

**Materials and Methods:**

The presence of *chxA* gene in 44 clinical *V. cholerae* NAG isolates were screened using PCR through specific primers designed for the receptor-binding domain (RBD) of *chxA* gene. The five PCR products of *chxA* gene were sequenced.

**Results:**

This study showed that *chxA* gene presented in 19 *V. cholerae* NAG isolates. The sequences analysis of 5 out of 19 the partial *chxA* genes amplicon showed that 4 of them belonged to *chxA* I and the other one belonged to *chxA* II subtypes. Two distinct clusters were revealed for these isolates by phylogenic analysis, too.

**Conclusion:**

The *chxA* gene contained high frequency among *V. cholerae* NAG isolates in Bushehr, Iran. The polymorphism study on RBD of cholixin gene is suggested as an appropriate method for phylogenic characterization of the various *chxA* gene subtypes.

## INTRODUCTION

A Gram-negative curved rod, *Vibrio cholerae*, is a member of the Vibrionaceae family (1). It has a monotrichous flagellum (H- antigen) and another surface antigen (O-antigen) (2). The current variation of O-antigen pattern results in the arrangement of 206 *V. cholerae* serogroups (3). *V. cholerae* strains belonging to O1 and O139 serogroups are related to epidemic and pandemic of cholera disease. Other members of other serogroups are recognized as *V. cholerae* non-O1/non-O139 strains (NAG or non-agglutinating *V. cholerae*) that exist in excess in the aquatic environment are associated with sporadic human infections (4). Several *V. cholerae* NAG strains are progressively being linked with human infection. These strains cause a wide clinical spectrum of gastroenteritis and extra-intestinal invasive disease including cholera-like disease, bloody diarrhea, ear and wound infection (more transmitted by seawater exposure) (5), meningitis, and bacteremia (predominantly in immunosuppressed patients) (6). Moreover, these serogroups seem to cause disease in their host and survive in an ecological niche by different strategies than O1 and O139 serogroups (7, 8).

Although the toxin coregulated pilus (TCP) and cholera toxin (CT) are characterized as the main common virulence factors of *V. cholerae* O1 and O139, most of the *V. cholerae* NAG strains are devoid of both factors. Nevertheless, genomic mining studies revealed, these serogroups may capture virulence factors through in horizontal gene transfer (HGT) mechanism and become toxic (6, 8, 9). Though the molecular mechanism of *V. cholerae* NAG strains pathogenicity are inadequate recognized (10), investigation of related virulence factors has drawn a great deal of attention, and so far various research investigations have revealed several virulence factors including variety of enterotoxins (NAG-ST, Shiga-like toxin) (11) hemagglutinin protease (HAP), *V. cholerae* protease (PrtV), hemolysin (HlyA), cytotoxin (RtxA), type III secretion system (TTSS) (7, 8, 12). Furthermore, it is demonstrated that utilizing an array of the various non-cholera toxin are the most important factors in pathogenicity and adaptive strategy of *V. cholerae* NAG strains.

Within mono-ADP-ribosyltransferase (mART) toxin family, cholix toxin (cholixin) is a new third member which is classified in the diphtheria toxin (DT) group. The cholixin *(V. cholerae)* along with exotoxin A *(P. aeruginosa)*, and diphtheria toxin *(C. diphtheria)* are specific for diphthamide residua in elongation factor II molecule resulting in the prevention of the protein synthesis of eukaryotic cell (12, 13). In addition, co-administration of cholixin and TNFα compared with the cholixin alone more enhanced the caspase activation, mitochondrial cytochrome release, poly-ADP-ribose fragmentation that result in cytotoxicity effects on human hepatocyte. Furthermore, research studies suggest that cholixin may be the main virulence factor that orchestrates with other virulence factors of *V. cholerae* NAG strains to increase the pathogenicity in humans (6, 14). However, it is stated that this toxin mediates significant intervention in the survival of the microorganism in an aquatic environment (10, 13).

The crystallography study of the full-length cholixin (71 kDa) demonstrates that this toxin involves tripartite domain structure. So that, their catalytic activity related to domain III obtained upon distraction of specific H-bonds to domain II by furin-like protease cleavage or reduction of the disulfide bond in the host cell. Furthermore, the other one and two domains take a part in receptor binding, membrane translocation respectively (13, 14). The *chxA* genes were grouped into three clusters (toxinotypes *chxA* I, II and III) up to now. There is not much information about the prevalence and genetic variety of the *chxA* gene among *V. cholerae* strains isolated from diarrheal patients and about their pathogenic mechanisms. The identification of frequency and genetic diversity of this exotoxin gives us novel visions of ChxA-mediated *V. cholerae* pathogenicity and the ChxA varied patterns may be linked to infections such as extra-intestinal infections (15).

The previous study showed that the cholix gene *(chxA)* was first recognized in environmental NAG strains of *V. cholerae* (9). As indicated by Purdy et al. (2010), the *chxA* gene was detected in less than 50% of NAG and about 15% of O1/O139 strains of *V. cholerae* isolated in littoral waters of southern California (16). In another investigation, the prevalence of *chxA* gene was reported less than 30% in *V. cholerae* NAG strains that produce cholixin (634-aa mature protein) with diverse cytotoxicity activity (12, 15).

The characterization of *chxA* gene has not been determined in Bushehr port as a tropical region of south-west of Iran. This study was aimed to define the frequency of *chxA* gene in clinical isolates of *V. cholerae* NAG strains in that region. In addition, the genetic diversity of *chxA* gene was further characterized using *chxA* sequencing.

## MATERIALS AND METHODS

### Ethics statement.

This research study was approved by the ethics committee of Bushehr University of Medical Sciences (code number: IR.BPUMS. REC.1394.133).

### Samples processing, bacteriology, and sero-grouping.

A total of 44 clinical *V. cholerae* NAG isolates were selected from the culture collection of the Department of Microbiology, School of Medicine, Bushehr University of Medical Sciences. The isolation period of isolates was included in a span of three years from 2013 to 2016. All of the clinical isolates were previously isolated from stool specimens and rectal swabs (in Cary-Blair transport medium) of diarrheal patients admitted to general hospitals of Bushehr University of Medical Sciences in the southwest of Iran. The identity of species and serogroups of all strains was confirmed by standard biochemical assays and their agglutinin activity with monospecific antibody to O1 and O139 serogroups. Consequently, all strains that had no agglutination reaction with O1 or O139 antisera were considered as *V. cholerae* NAG (non-O1/non-O139) strains (17). All isolates were stored in 30% glycerol stock at −70°C following by culturing in brain heart infusion broth and subsequently on thiosulfate-citrate-bile salts-sucrose (TCBS) agar at 37°C when required (18).

### DNA template preparation.

The genomic DNA template was extracted from an overnight culture of *V. cholerae* NAG strains using Exgene Cell SV kit (GeneAll, South Korea) according to the manufacturer’s directions. Subsequently, isolated DNA was diluted 100-fold in sterile ultrapure water and 2 μl of the diluted genomic DNA was used in a final volume of 25 μl PCR reaction. However genomic DNA and amplified PCR products were analyzed by agarose gel electrophoresis in TAE buffer (Tris-acetate 40 mM, EDTA 1 mM, [pH 8.5]) at 75V around 40 minutes by using 1.5% and 2% agarose gel respectively (10). The precast agarose gel was contained 1 μL/ml of 10 fold diluted of stock DNA fluorescent staining dye (DS1000/SMOBIO). Images of agarose gel electrophoresis results were captured under UV light photography by using a gel documentation system (BioDoc-It, Bio-Rad Laboratories Inc.) (19).

### PCR amplification of *chxA* gene.

The detection of the partial *chxA* gene was performed by PCR. The sequence of the forward (TGGTGAAGATTCTCCTGCAA) and reverse (CTTGGAGAAATGGATGCGCTG) primers of the partial *chxA* gene were reported previously (20) and were obtained from Macrogen (South Korea) Biotechnology Company. In addition, Taq DNA polymerase Master Mix RED and the *Pfu* DNA polymerase were provided by Amplicon (Danish) and ViVantis (Malaysia) Biotechnology Companies, respectively. All PCR amplifications were performed in a thermal cycler (T100, Bio-Rad). To evaluate the effect of annealing temperature on the bias affected by primer mismatches, the gradient PCR was experienced at annealing temperature ranges from 54 to 63°C to optimize the annealing temperature of *chxA* primers. Then, two different protocols were used to prepare the PCR amplification mixture. In order to detect *chxA*, PCR reagents were prepared in a final volume of 25 μL which included, 2 μL of diluted genomic DNA template, 1 μL of each primer (10 pmol/μL), 10 μL of master mix 2×, and 11 μL of sterile ultrapure water (10). For sequencing of amplicons, PCR reagents were prepared in a final volume of 25 μL containing 2 μL of diluted genomic DNA template, 1 μL of each primer (10 pmol/μL), 2.5 μL of 10× buffer A, 0.3 μL of *pfu* DNA polymerase (5 U/μL), 1 μL of dNTP mixture (10 mM each), 0.75 μL of MgCl_2_ (50 mM) and 16.5 μL of sterile ultrapure water. Thermal cycling of the amplification mixtures were consisted of initial denaturation at 94°C for 10 min followed by 30 cycles of denaturation at 94°C for 30 seconds, primer annealing at 62.3°C (Optimum temperature) for 30 seconds and extension at 72°C for 30 seconds and followed by a final extension at 72°C for 5 min. All experiments were done in duplicate (21).

### Nucleotide sequencing and analysis.

In the present study, five PCR amplicons specific for partial *chxA* genes (421 bp) detected in the clinical *V. cholerae* NAG isolates were randomly selected. Then their sequencing analyses were performed by the sequencing service of Macrogen Company (South Korea). Both 5/ and 3/ ends of each PCR amplicon were sequenced with the same primers used to amplify the region (22).

### Bioinformatics analysis.

The sequences of the primers specific to the *chxA* gene were confirmed by submission to NCBI server and related amplicon size was predicted by primer blast service (https://www.ncbi.nlm.nih.gov/tools/primer-blast). In addition, the *chxA* amplicon sequencing results were initially edited using Chromas program version 1.45 and Gene Quest DNASTAR Inc software. The 421 bp encoding as a part of the *chxA* gene of five clinical *V. cholerae* NAG isolates (V2, V19, V25, V38, V45) were submitted to NCBI server to find the similarity between the sequences and compare the query nucleotide sequences to sequence database and calculate the statistical significance using nucleotide Blast (https://blast.ncbi.nlm.nih.gov/Blast.cgi). The genetic diversity was analyzed using multiple alignment Clustal Omega programs (https://www.ebi.ac.uk/Tools/msa/clustalo) in the EBI server to generate a phonogram and a neighbor-joining tree was constructed, according to maximum likelihood method (10, 23).

### Nucleotide sequence accession numbers.

The partial nucleotide sequences of the *chxA* genes have been deposited in the DNA Data BankIt with accession numbers MH801211 to MH801214 and MH793270.

## RESULTS

### Frequency of *chxA* gene in clinical *V. cholerae* NAG isolates.

**Fig. 1.**
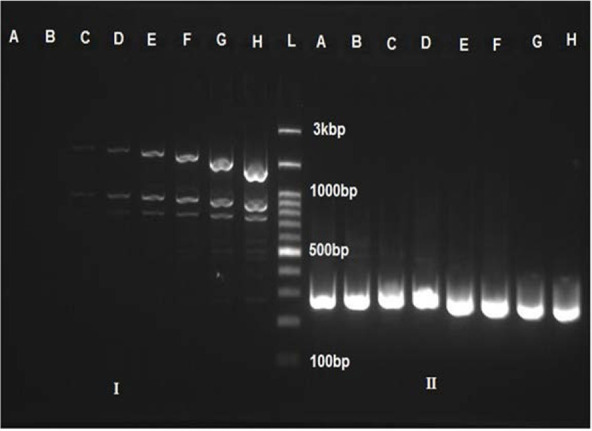
Agarose gel electrophoresis (2%) of gradient PCR amplicon of *chxA* primers. Annealing temperatures from A to H are 63, 62.3, 61.2, 59.5, 57.5, 55.7, 54.6, 54 0C, respectively. L: DM2300 DNA ladder, I (V6) and II (V10) are clinical *V. cholerae* NAG isolates.

A total of 44 clinical *V. cholerae* NAG isolates, were screened by PCR for the presence of the partial *chxA* gene. The *chxA* primers identified their specific complementary targets in the genomic DNA template at optimum annealing temperature 62.3 (Fig. 1). Among all isolates, 19 (43.1%) of *V. cholerae* NAG strains harbored the *chxA* gene.

### The diversity of *chxA* gene in clinical *V. cholerae* NAG isolates.

**Fig. 2.**
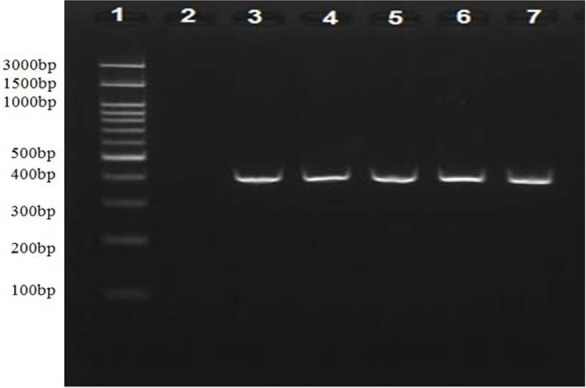
Agarose gel electrophoresis (2%) of PCR amplification using *pfu* DNA polymerase targeted to partial *chxA* gene (421 bp). 1: DM2300 DNA ladder, 2: (Negative control), 3–7 show *chxA* PCR amplicon related to different clinical *V. cholerae* NAG isolates.

**Fig. 3.**
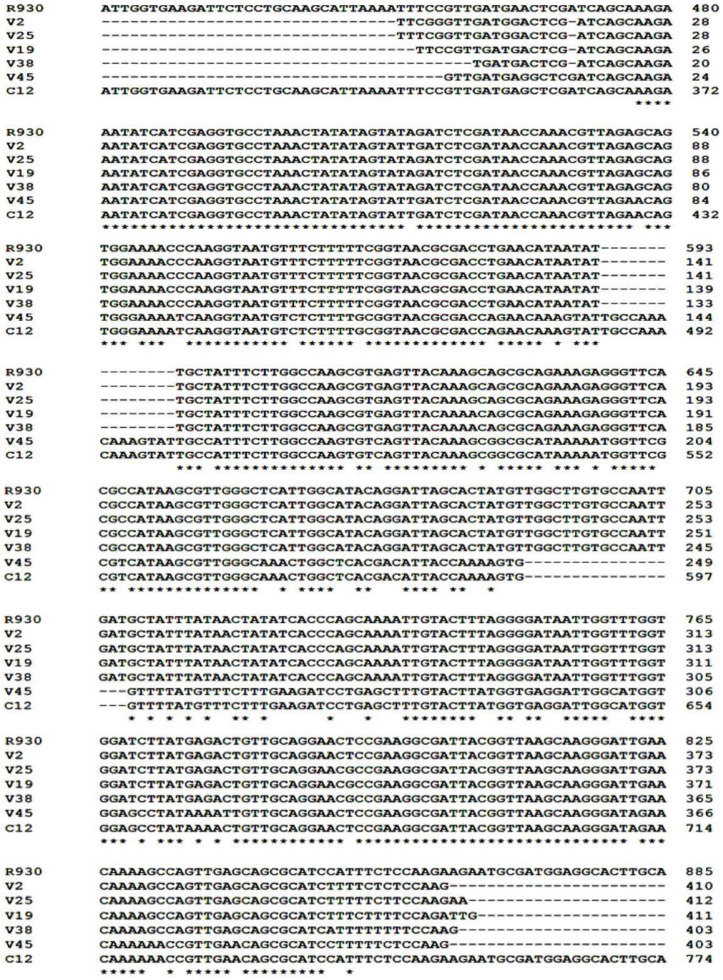
Multiple Clustal Omega alignments of partial *chxA* gene sequence fragments of clinical *V. cholerae* NAG isolates along with published the full-length *chxA* sequences from C12 and R930 subtypes. Common nucleic acids in all sequences are designated with an asterisk.

**Fig. 4.**
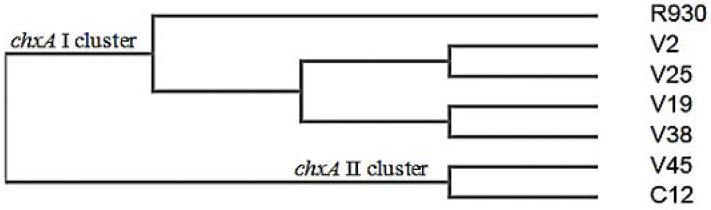
Phylogeny of partial *chxA* genes from clinical *V. cholerae* NAG isolates. The phenogram is designed by unweighted pair group method analysis (UPGMA) of the Clustal Omega Program.

The five partial *chxA* gene sequences (Fig. 2, 421 bp) of all clinical *V. cholerae* NAG isolates harbored this gene (n=19) were aligned and compared with the published sequence databases of the *chxA* gene (Fig. 3). However, the partial *chxA* gene sequence of V45 isolate showed maximum identity (99%) with the published *chxA* gene sequence of C12 subtype (GenBank accession no. GU299628.1) and minimum identities (79%) with *chxA* gene sequence of R930 subtype (GenBank accession no. KR259136.1). Conversely, the partial *chxA* gene sequence of V2 isolate showed maximum identity (99%) with the published *chxA* gene sequence of R390 subtype and minimum identity (79%) with *chxA* gene sequence of C12 subtype. The results of pair wised comparison showed, the partial *chxA* gene sequence of V19, V25, V38 isolates possessed the maximum identities 97.8%, 98.8%, 98.3% with the same target of V2 isolate respectively, but the partial *chxA* gene sequence of V45 isolate showed the minimum identity (75.7%) with the partial *chxA* gene V2 isolate. Furthermore, based on the general similarity of partial *chxA* gene sequences, the phylogenetic analysis (Fig. 4) showed that the five partial *chxA* genes were differentiated into five subtypes and more grouped into two main *chxA* clusters I (n=4) and II (n=1). The sequences of *chxA* cluster I and *chxA* cluster II are close to the published R930 (GenBankaccession no. KR259136.1) and C12 (Gen-Bank accession no. GU299628.1) subtype sequences, respectively.

## DISCUSSION

Although *V. cholerae* NAG strains do not produce cholera toxin, they may capture virulence factors through a horizontal gene transfer (HGT) route and become toxic (8, 11, 13). Among a variety of toxins investigated in *V. cholerae* serogroups, cholixin possesses a major role in pathogenesis and environmental compatibility (14, 16). Cholixin which is recently identified as a new potent ADP ribosyltransferase inhibiting protein synthesis of the eukaryotic cell, is globally found in clinical and environmental *V. cholerae* NAG isolates (12, 13, 24, 25). Furthermore, according to Awasthi et al. (2013), three novel variants of cholixin included ChxA I, ChxA II, and ChxA III have been discovered previously (10). They argue that the ChxA I, ChxA II variants may stimulate extra-intestinal infections and ChxA II can be more lethal than ChxA I in mice (15). Therefore, as the prevalence of *chxA* gene variants is an important significance in ecological and epidemiological properties of *V. cholerae* NAG strains (16), we have used a PCR method to identify the frequency of *chxA* gene in clinical *V. cholerae* NAG strains using specific primers targeted the receptor-binding domain (RBD) of the cholixin in this study. Our results indicate that the *chxA* gene presents among more than 40 percent of *V. cholerae* NAG isolates of clinical origin. The results provide more evidence for previous studies indicated various prevalence rates of *chxA* gene in the different geographical regions (26). For example, Purdy et al. in a study conducted on determining the global prevalence of *chxA* gene in clinical and environmental *V. cholerae* strains showed that approximately less than 50 percent of *V. cholerae* NAG strains harbored the *chxA* gene (16) as well as a *chxA* gene frequency of nearly 17 percent was demonstrated among clinical *V. cholerae* non-O1/non-O139 isolated from German and Austrian patients (23). Indeed, it was stated by Awasthi et al. that the presence of *chxA* gene has no dependency on the occurrence of other virulence genes among *V. cholerae* strains. This finding along with the high distribution of *chxA* gene in various clinical and environmental *V. cholerae* NAG isolates revealed the potential of cholixin as the main virulence factor in NAG strains of *V. cholerae* (13, 24). The *V. cholerae* NAG strains used in this study were isolated in Bushehr, shows the incidence of the *chxA* gene in *V. cholerae* strains originating from Iran. Even though the role of cholixin to create the disease in humans is not clear, our result is in agreement with the recent diarrhea outbreaks in Kenya caused by *V. cholerae* NAG strain that harbored the *chxA* gene (27). On the other hand, cholixin is a versatile protein in *V. cholerae* strains as the aquatic organism that may employ the toxin as a colonization factor to comfort mutualistic interaction between *V. cholerae* and aquatic organisms. This behavior may result in the protection of environmental *V. cholerae* strains against peripheral stress (13, 20). It is stated that the expression of *chxA* is related to a niche condition in which the *V. cholerae* strains are located there. Therefore, it is more probable that the role of the *chxA* gene in the development of animal infection is not correlated to survive in the environment directly (20).

Another purpose of this study was to determine the genetic diversity and toxinotype of *chxA* genes that were further characterized by using the partial *chxA* gene sequencing. The sequencing of PCR amplicons targeting *chxA* gene partially provided a reproducible and precise method for identifying the various subtype of *chxA* gene in *V. cholerae* strains. The sequences analysis of 5 out of 19 the partial *chxA* genes identified in *V. cholerae* NAG during our study showed that 4 and 1 belonged to *chxA* I and *chxA* II subtypes, respectively. The dominant presence of the *chxA* I subtype in our study (4 out of 5 ∼79 %) is in agreement with the findings of Awasthi et al. (2014) (10) but they performed PCR-RFLP assay for characterizing three subtypes of the entire *chxA* gene in *V. cholerae* and found out that the *chxA* I subtype (33 out of 42, ∼79%) was predominant. To concur with the previous study (10), the *chxA* III subtype was not detected in our study. However, due to the sequencing limitation sample, we are unable to rule out the occurrence of this gene subtype in all *V. cholerae* NAG isolates tested. The phylogenetic analysis reveals that there are high sequences of diversity in *chxA* sequences tested (Five subtypes among five partial *chxA* sequences). Accordingly, it seems the partial *chxA* gene encoded receptor-binding domain (RBD) of cholixin contains gene polymorphisms, which may be appropriate for identification and phylogenic characterization of the various *chxA* gene subtypes. The comparison of the partial *chxA* sequences tested in this study with the three different subtypes of *chxA* (I, II, III) reported by Awasthi et al. (2013) (15) showed that the V2, V19, V25, and V38 partial *chxA* subtypes have the most sequence identities (99%) with the entire *chxA* I gene against other *chxA* II (81%) and *chxA* III (92%) subtypes. Moreover, the V45 partial *chxA* subtype has the most sequence identity (91%) with the entire *chxA* II gene against other *chxA* I (78%) and *chxA* III (79%) subtypes (Data not have shown).

It is indicated that unlike with recombinant ChxA III (rChxA III), rChxA I and rChxA II toxinotypes expressed different cytotoxic effects on various eukaryotic cells (24, 28). There is compelling evidence that amino acid variations in the RBD of various cholixin subtypes reflect on their receptor recognition and their effects on various host cells. As indicated by Awasthi et al. (2013), recombinant ChxA I and ChxA III toxins feasibly possess the same receptor on HeLa cell whereas ChxA II could not be attached to the shared receptor. They suggested that various cytotoxicity effects of ChxA II could be due to amino acid variation in their RBD, expression of different receptors on various cell hosts or occurrence of another mechanism to target host cells. Also, they explained that there was no significant correlation between the failure of the cytotoxicity effect of recombinant ChxA III and binding feasibility or catalytic activity because this toxinotype could inhibit the induced cytotoxicity effect of ChxA I (15).

Although the results of the study performed on the rabbit ileal loop showed all ChxA toxinotypes possesses no enterotoxicity effect, the results of the experience conducted by systemic injection (intravenous and intraperitoneal) of rChxA toxinotypes revealed that both ChxA I and II toxinotypes could cause lethal damage to internal organ of mice, especially the liver (15). At least in the animal model, it was concluded that these toxinotypes may be related to extra-intestinal infections (12).

## CONCLUSION

In conclusion, this study has shown the high prevalence of various subtypes of the *chxA* genes in clinical *V. cholerae* NAG isolates residents in this geographical region. To the best of our knowledge, this is the first report on the existence of two *chxA* subtype genes (*chxA* I, *chxA* II) from Iran. Among them, the *chxA* I subtype gene is predominant. The small sample size of sequencing experiments may be the reason why the *chxA* III subtype gene is not identified and it could count as one of the limitations for our study. Our results reveal that the PCR product sequencing of the RBD is a simple and precise method for evaluation of the genomic diversity of *chxA* genes of *V. cholerae.* Considering the importance of *chxA* gene in increasing virulence potential of *V. cholerae*, more broadly research is also needed to determine the variety of clinical and environmental sources for the presence of the isolates harbored this gene. The important advantages of these results are helping to design a better prevention program to control these strains and improve the perception of the role of *chxA* gene in the pathogenicity of *V. cholerae* in the future.
